# Physiological functions of endoplasmic reticulum stress transducer OASIS in central nervous system

**DOI:** 10.1007/s12565-013-0214-x

**Published:** 2013-11-16

**Authors:** Atsushi Saito

**Affiliations:** Department of Biochemistry, Institute of Biomedical and Health Sciences, University of Hiroshima, 1-2-3 Kasumi, Minami-ku, Hiroshima, 734-8553 Japan

**Keywords:** ER stress, Unfolded protein response, OASIS, Astrocyte differentiation, Gcm1

## Abstract

Eukaryotic cells can adapt to endoplasmic reticulum (ER) dysfunction by producing diverse signals from the ER to the cytosol or nucleus. These signaling pathways are collectively known as the unfolded protein response (UPR). The canonical branches of the UPR are mediated by three ER membrane-bound proteins: double-stranded RNA-dependent protein kinase (PKR)-like endoplasmic reticulum kinase (PERK), inositol-requiring enzyme-1 (IRE1) and activating transcription factor 6 (ATF6). These ER stress transducers basically play important roles in cell survival after ER stress. Recently, novel types of ER stress transducers that share a region of high sequence similarity with ATF6 have been identified. They have a transmembrane domain, which allows them to associate with the ER, and possess a transcription-activation domain and a basic leucine zipper (bZIP) domain. These membrane-bound bZIP transcription factors include OASIS, BBF2H7 CREBH, CREB4 and Luman, and are collectively referred to as OASIS family members. Despite their structural similarities with ATF6, differences in activating stimuli and tissue distribution indicate specialized functions of each member on regulating UPR signaling in specific organs and tissues. One of them, OASIS, is expressed preferentially in astrocytes in the central nervous system (CNS). OASIS temporally regulates the differentiation from neural precursor cells into astrocytes to promote the expression of Glial Cell Missing 1 through dynamic interactions among OASIS family members followed by accelerating demethylation of the *Gfap* promoter. This review is a summary of our current understanding of the physiological functions of OASIS in the CNS.

## ER stress and canonical unfolded protein response

**Fig. 1:**
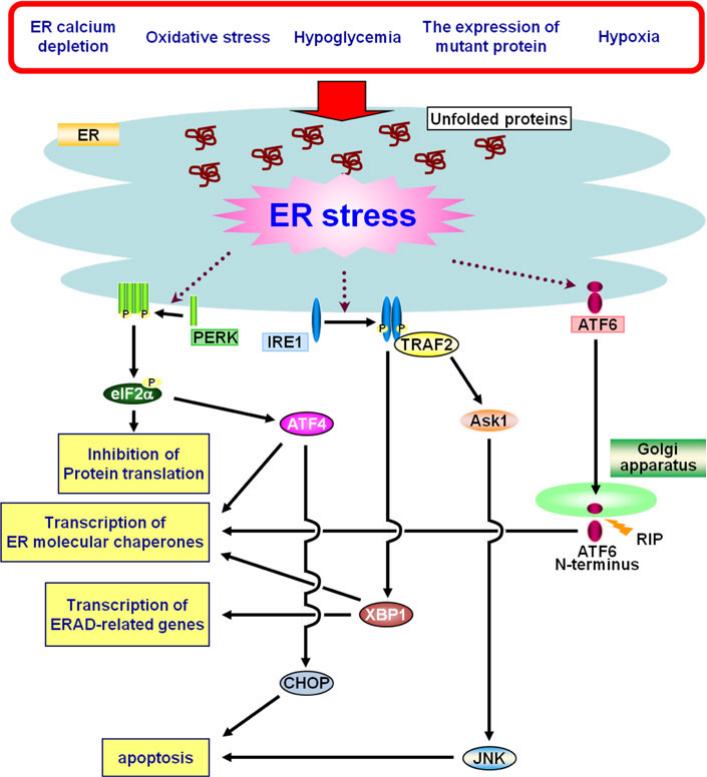
Schema of endoplasmic reticulum (ER) stress and unfolded protein response (UPR) signaling. Activation of the UPR leads to a transient translational inhibition, followed by the activation of transcription of ER molecular chaperones and the degradation of the unfolded proteins accumulated in the ER [ER-associated degradation (ERAD)]. If these strategies fail, cells undergo ER stress-induced apoptosis. *Ask1* apoptosis signal-regulating kinase 1, *ATF4* activating transcription factor 4, *CHOP* C/EBP homologous protein, *JNK* c-jun N-terminal kinase, *RIP* regulated intramembrane proteolysis, *TRAF2* TNF receptor-associated factor 2, *XBP1* X-box binding protein 1, *P* phosphorylation

The endoplasmic reticulum (ER) is an essential organelle that regulates the synthesis and post-translational modifications of secretory and membrane proteins. Nascent proteins are folded in the ER with the assistance of molecular chaperones and folding enzymes. Various pathophysiological conditions, such as ER-calcium depletion, oxidative stress, hypoglycemia, expression of mutated proteins and hypoxia, interfere with the correct folding of proteins followed by promoting the accumulation of these unfolded or misfolded proteins in the ER lumen. These conditions are collectively termed ER stress, and have the potential to induce cellular damage. The ER responds to these perturbations by activating an integrated signal transduction pathway, called the unfolded protein response (UPR) (Ron 2002; Kaufman 2002; Schroder and Kaufman 2005). Activation of the UPR leads to a transient translational inhibition, followed by the activation of transcription of ER molecular chaperones and the degradation of unfolded proteins accumulated in the ER [ER-associated degradation (ERAD)]. In mammalian cells, ER stress-sensing and UPR signaling are regulated by three well-established ER stress transducers: double-stranded RNA-dependent protein kinase (PKR)-like endoplasmic reticulum kinase (PERK) (Harding et al. 1999), inositol-requiring enzyme-1 (IRE1) (Tirasophon et al. 1998; Urano et al. 2000), and activating transcription factor 6 (ATF6) (Li et al. 2000; Yoshida et al. 2000). PERK directly phosphorylates the α subunit of eukaryotic initiation factor (eIF2α) and leads to shut-down of most cellular protein synthesis (Harding et al. 2000). IRE1, ATF6 and ATF4, which is downstream of PERK-eIF2α pathway, are involved in transcriptional regulation of ER molecular chaperones and ERAD-related genes. If these strategies fail, cells undergo ER stress-induced apoptosis (Ron 2002; Kaufman 2002; Schroder and Kaufman 2005). Thus, cell fates after ER stress are determined by the balance of cell survival and death signals regulated by the UPR (Fig. 1).

## Novel types of membrane-bound basic leucine zipper transcription factors activated by regulated intramembrane proteolysis

**Fig. 2:**
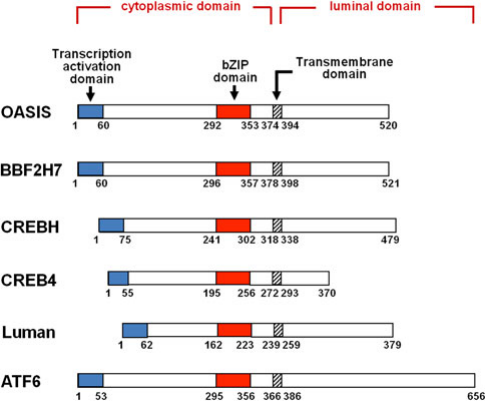
Predicted peptide features of mouse OASIS, BBF2H7, CREBH, CREB4, Luman and ATF6. Novel types of ER stress transducers, OASIS family members including OASIS, BBF2H7, CREBH, CREB4 and Luman share a region of high sequence similarity with ATF6. They have a transmembrane domain, which allows them to associate with the ER, and possess a transcription-activation domain and a bZIP domain

In humans, there are more than 55 known basic leucine zipper (bZIP) transcription factors (Newman and Keating 2003). By sequence similarity in the coiled-coil region, these transcription factors can be divided into 16 different families. Recently, novel types of ER stress transducers, i.e., ER membrane-bound bZIP transcription factors that share a region of high sequence similarity with ATF6, have been identified. They have a transmembrane domain that allows them to associate with the ER, and possess both a transcription-activation domain and a bZIP domain. The new types of ER stress transducers include OASIS (Kondo et al. 2005; Murakami et al. 2009), BBF2H7 (Kondo et al. 2007; Saito et al. 2009), CREBH (Omori et al. 2001; Zhang et al. 2006), CREB4 (Qi et al. 2002; Cao et al. 2002; Adham et al. 2005; Nagamori et al. 2005), and Luman (Lu et al. 1997; DenBoer et al. 2005; Liang et al. 2006) (Fig. 2). Interestingly, each of them commonly contains the consensus sequence for cleavage by site-1 protease (S1P) and site-2 protease (S2P) in its luminal segment, indicating that they are processed at transmembrane regions by regulated intramembrane proteolysis (RIP) (Ye et al. 2000; Bailey and O’Hare 2007). Their cleaved N-terminal fragments translocate into the nucleus to act as transcription factors. In this review, these bZIP transmembrane transcription factors are referred to as OASIS family members. Despite structural similarities among these proteins and ATF6, differences in activating stimuli and tissue distribution indicate that these proteins play specific roles in regulating the UPR signaling in specific organs and tissues. OASIS, BBF2H7, CREBH, and CREB4 are expressed preferentially in osteoblasts and astrocytes (Kondo et al. 2005; Murakami et al. 2009), chondrocytes (Saito et al. 2009), liver cells (Omori et al. 2001), and prostate and testis (Cao et al. 2002), respectively. The Luman transcript is present in a wide range of adult and fetal tissues (Lu et al. 1997), but its translated product has been found only in trigeminal ganglional neurons and monocytes, and dendritic cells (DCs) (Lu and Misra 2000; Ko et al. 2004; Eleveld-Trancikova et al. 2010). Evolutionarily, orthologues of ATF6 and OASIS family proteins exist in organisms higher than* Caenorhabditis  elegans*. *C. elegans* contains three bZIP transcription factors. Although the degree of homology is less than those in the mammalian family, these proteins are related most closely to ATF6, OASIS and Luman (Shen et al. 2005; Fox et al. 2010). In *Drosophila*, two bZIP transcription factors are related most closely to ATF6 and OASIS (Fox et al. 2010). In vertebrates, cells are diversely differentiated to play proper roles for various biological phenomena, and also adapt to environmental parameters. Vertebrate cells adjust the functionality and capacity of their ER depending on the diversity of cell types. Thus, the signaling of the vertebrate UPR, which is regulated by canonical ER stress transducers and OASIS family members, has considerable complexity and is highly developed in cell-type specific patterns. This review is focused on the current understanding of the biological characteristics and physiological functions of OASIS in the central nervous system (CNS).

## Structure and activation mechanisms of OASIS

**Fig. 3:**
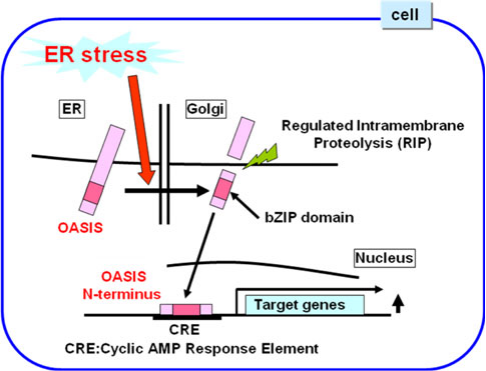
Activation mechanisms of OASIS. OASIS is cleaved in response to ER stress at the transmembrane region by regulated intramembrane proteolysis (RIP), and the N-terminus containing bZIP domain translocates into the nucleus to promote expression of target genes through the binding to the CRE sequence

The *Oasis* gene was originally identified as a gene specifically induced in long-term cultured astrocytes (Honma et al. 1999). The OASIS protein is a bZIP transcription factor of the cyclic AMP-response element (CRE)-binding protein (CREB)/ATF family, with a transmembrane domain that allows it to associate with the ER. Its N-terminus containing the transmembrane domain is 31 % identical to ATF6, but its C-terminus, which is within the ER lumen, does not show homology to ATF6. In the luminal segment, OASIS contains the sequence RSLL (beginning at residue 423), which fits the RxxL consensus for S1P whose active site faces the Golgi lumen. Indeed, under ER stress conditions, OASIS is cleaved at the site by S1P and subsequently at the transmembrane domain by S2P (Kondo et al. 2005; Murakami et al. 2006). The cleaved N-terminus containing transcription-activation domain and bZIP domain translocates into the nucleus to act as a transcription factor through the binding to the CRE sequence (Fig. 3). ATF6 contains a stretch of amino acid sequence in its luminal domain that is essential for translocation from the ER to the Golgi apparatus (Shen et al. 2002). However, translocation to the Golgi apparatus and proteolytic processing are intact in all deletion mutants for the luminal domain of OASIS, indicating that OASIS does not have significant sequences for Golgi localization signaling (Murakami et al. 2006). Therefore, the translocation system from the ER to the Golgi apparatus is quite different between OASIS and ATF6 under ER stress conditions. Under normal conditions, ATF6 is bound constitutively by an ER-resident chaperone BiP in its luminal domain and rendered inactive (Shen et al. 2002; Chen et al. 2002). Accumulation of unfolded proteins in the ER results in the dissociation of BiP from the luminal domain of ATF6 and then ATF6 is translocated to the Golgi apparatus. The ER luminal domain of OASIS does not possess the sensing function for unfolded proteins. OASIS is degraded easily under normal conditions via the ubiquitin–proteasome pathway (Kondo et al. 2012). An ER stress condition enhances the stability of OASIS. The stabilized OASIS is transported automatically from the ER to the Golgi apparatus and is then cleaved to generate N-terminus by RIP. Thus, the ER stress-sensing function is not necessary in the ER luminal domain of OASIS for its activation.

## Physiological functions of OASIS in skeletal systems

*Oasis* is highly expressed in osteoblasts of osseous tissues (Murakami et al. 2009) and astrocytes of the CNS (Kondo et al. 2005; Chihara et al. 2009), and to considerable levels in intestine, salivary glands, and prostate (Honma et al. 1999; Omori et al. 2002; Asada et al. 2012). In C6 glioma cell lines and primary cultured osteoblasts, *Oasis* is induced at the transcriptional level after treatment with ER stressors such as tunicamycin or thapsigargin (Kondo et al. 2005). *Oasis* deficient (*Oasis* ^−/−^) mice are born at the expected Mendelian ratios, but the mice exhibit severe osteopenia involving a decrease in bone density at all skeletal sites and spontaneous fractures (Murakami et al. 2009). From detailed analyses on gene expression in *Oasis* ^−/−^ bones, *Type I collagen* (*Col1*)—a major component of osseous tissues—was identified as one of the targets for OASIS. OASIS activates the transcription of *Col1* through direct binding to a CRE-like sequence that exists in the *Col1* promoter regions. Indeed, *Oasis* ^−/−^ osseous tissues show a significant decrease in the amounts of type I collagen. The defect of bone formation involving decreased amounts of type I collagen is rescued completely by osteoblast-specific overexpression of OASIS, indicating that osteopenia in *Oasis* ^−/−^ mice is caused primarily by deletion of *Oasis* and its target, *Col1* gene in osteoblasts (Murakami et al. 2010).

## *Oasis*^−/−^ mice exhibit decreased numbers of astrocytes

**Fig. 4:**
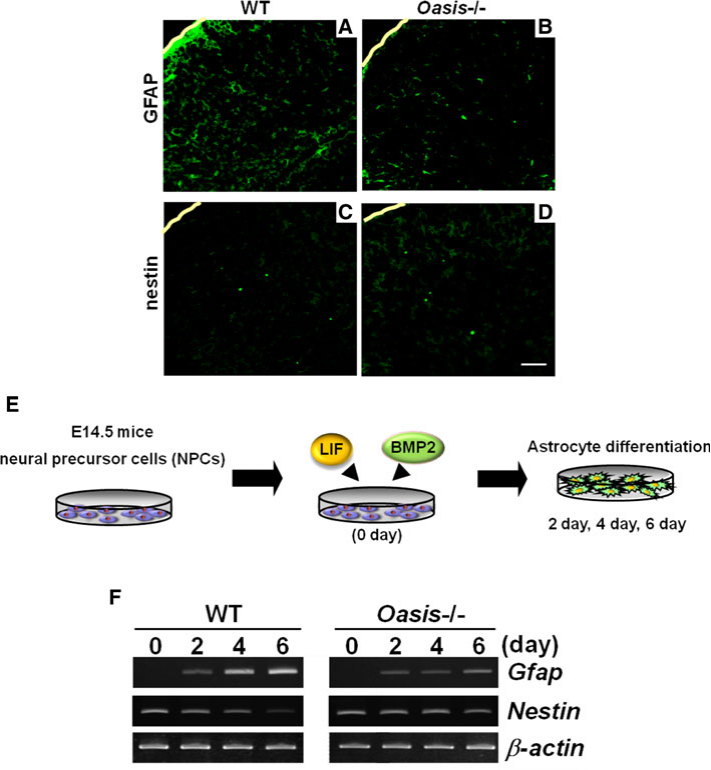
Differentiation of neural precursor cells (NPCs) into astrocytes is delayed in *Oasis* ^−/−^ mice. Immunohistochemical analysis of glial fibrillary acidic protein (GFAP) (**a**, **b**) and nestin (**c**, **d**) in the cerebral cortices of E18.5 WT and *Oasis* ^−/−^ mice. The number of GFAP-positive cells was lower, and that of nestin-positive cells was higher in *Oasis* ^−/−^ mice. *Yellow lines* Surfaces of cerebral cortices.* Bar* 100 μM. **e** Primary cultured NPCs were prepared from the telencephalons of E14.5 WT and *Oasis* ^−/−^ mice. Cells were treated with leukemia inhibitory factor (LIF) and bone morphogenetic protein 2 (BMP2) to promote the differentiation of NPCs into astrocytes. **f** RT-PCR analysis of *Gfap* and *Nestin* in primary cultured NPCs treated with LIF and BMP2 for the indicated times. The up-regulation of *Gfap* and the down-regulation of *Nestin* are inhibited in *Oasis* ^−/−^ cells

As mentioned above, *Oasis* is also highly expressed in astrocytes of the CNS. OASIS is up-regulated in reactive astrocytes after neuronal degeneration induced by kainic acid (KA). In hippocampal astrocytes injured by intraperitoneal injection of KA, ER stress is induced and *Oasis* mRNA is strongly up-regulated (Chihara et al. 2009). Pyramidal neurons in the hippocampi of *Oasis* ^−/−^ mice are more susceptible to the toxicity induced by KA than those of wild-type (WT) mice. The number of glial fibrillary acidic protein (GFAP)-positive reactive astrocytes, an astrocyte marker, was decreased in the hippocampi of *Oasis* ^−/−^ mice compared with those of WT mice after brain injury. In embryonic stages, *Oasis* mRNA is barely observed in the cerebral cortices of embryonic day (E) 14.5 mice; however, strong signals are detected at E16.5 and E18.5 (Saito et al. 2012), coinciding with the initiation of extensive differentiation of neural precursor cells (NPCs) into astrocytes. *Oasis* ^−/−^ mice exhibit impaired astrocyte differentiation in embryonic stages. The numbers of cells positive for GFAP are significantly higher in the cerebral cortices of WT mice than in those of *Oasis* ^−/−^ mice at E18.5 (Fig. 4a, b) (Saito et al. 2012). By contrast, those of nestin—an NPC marker—are higher in the cerebral cortices of *Oasis* ^−/−^ mice than in those of WT mice at E18.5 (Fig. 4c, d). The impaired astrocyte differentiation is also observed in primary cultured NPCs prepared from E14.5 *Oasis* ^−/−^ mice telencephalons. In primary cultured *Oasis* ^−/−^ NPCs treated with leukemia inhibitory factor (LIF) and bone morphogenetic protein 2 (BMP2), which promote the differentiation of NPCs into astrocytes (Bonni et al. 1997; Nakashima et al. 1999a, b) (Fig. 4e), the induction of *Gfap* expression and the reduction of *Nestin* expression are significantly inhibited compared with those of WT cells (Fig. 4f).

## The target of OASIS in NPCs is Gcm1

**Fig. 5:**
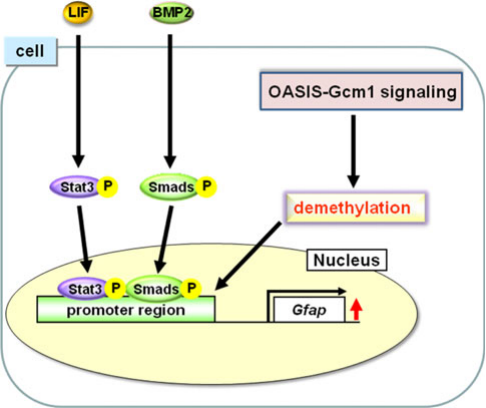
OASIS-Gcm1 signaling is involved in demethylation of the *Gfap* promoter. Activation of Stat3 and Smads, and epigenetic regulation of the methylation status of the *Gfap* promoter, are essential for differentiation of NPCs into astrocytes. Stat3 and Smads are phosphorylated downstream of LIF and BMP2, and phosphorylated Stat3 and Smads bind to demethylated sites in the *Gfap* promoter to promote transcription of *Gfap*. OASIS-Gcm1 signaling does not alter the levels of phosphorylated Stat3 and Smads, but regulates the demethylation status of the *Gfap* promoter region

Glial Cell Missing 1 (Gcm1) is identified as a target of OASIS. OASIS promotes the expression of *Gcm1* through the binding to the CRE-like sequence in the promoter region of *Gcm1* in CNS and trophoblasts (Schubert et al. 2008; Saito et al. 2012). The *Gcm1* *Drosophila* ortholog, *gcm*, has been reported to control neuronal and glial fates (Hosoya et al. 1995; Jones et al. 1995; Vincent et al. 1996). In mammalian cells, the introduction of Gcm1 into cultured brain cells derived from mice induced the astrocyte lineage (Iwasaki et al. 2003). As described above, Gcm1 is critical for astrocyte differentiation; however, the detailed mechanisms regulating differentiation have not been defined. It is well known that the activation of Stat3 and Smads, and epigenetic regulation of the methylation status of the *Gfap* promoter, are essential for the differentiation of NPCs into astrocytes. Phosphorylated-Stat3 and Smads bind to demethylated sites in the *Gfap* promoter to form a Stat3-Smads-p300 complex and promote transcription of *Gfap* (Bonni et al. 1997; Nakashima et al. 1999a, b, Takizawa et al. 2001). Although the levels of phosphorylated Stat3 and Smads are not changed, the amount of demethylation of the *Gfap* promoter is decreased significantly in *Oasis* ^−/−^ NPCs compared with those of WT NPCs (Saito et al. 2012). Thus, the delayed astrocyte differentiation in *Oasis* ^−/−^ cells is caused by inhibition of demethylation of the *Gfap* promoter (Fig. 5). The mammalian homologs of *gcm* are *Gcm1* and *Gcm2*. These molecules show little homology and have no common functional domains except for the GCM-motif (Akiyama et al. 1996). Previous report have shown that both have the potential to accelerate demethylation of the *Hes5* promoter by direct binding to the GCM-binding site in the promoter followed by acquiring stem cell properties, but *Gcm2* is a more crucial factor than *Gcm1* for demethylation of the *Hes5* promoter because of the lower expression of *Hes5* in *Gcm2* ^−/−^ mice than in *Gcm1* ^−/−^ mice (Hitoshi et al. 2011). The expression of *Gcm1* is significantly up-regulated at later stages of mouse embryonic development, whereas that of *Gcm2* is transiently up-regulated at the early stage (Hitoshi et al. 2011). Therefore, we presume that *Gcm2* is involved mainly in demethylation of the *Hes5* promoter at the early stage of mouse embryonic development and, conversely, that *Gcm1* mainly plays a role in the demethylation of the *Gfap* promoter in the late stage. The distinct expression patterns and target promoters for demethylation between *Gcm1* and *Gcm2* may determine glial and neuronal cell lineages, respectively.

## Gcm1 expression modulation by OASIS family members

**Fig. 6:**
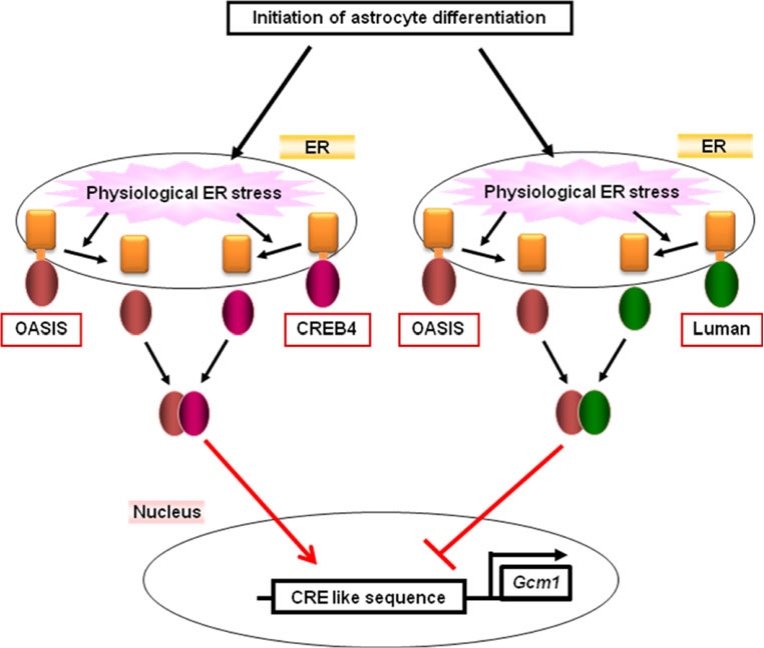
OASIS, CREB4 and Luman N-termini form heterodimers to regulate Gcm1 expression. OASIS-CREB4 heterodimers activate transcription of *Gcm1* during astrocyte differentiation. Conversely, the Luman N-terminus inhibits the formation of OASIS-CREB4 heterodimers by binding to the OASIS N-terminus, down-regulating transcription of *Gcm1*

The bZIP type transcription factors including OASIS family members are known to form homodimers or heterodimers to promote transcription (Hai and Hartman 2001; Zhang et al. 2006). During astrocyte differentiation, not only OASIS but also CREB4 and Luman regulate the transcription of *Gcm1* (Saito et al. 2012). OASIS and CREB4 N-termini form heterodimers and activate transcription of *Gcm1* during astrocyte differentiation. Conversely, the Luman N-terminus inhibits the formation of the OASIS-CREB4 heterodimer by binding to the OASIS N-terminus and down-regulating transcription of *Gcm1*, leading to a cessation of astrocyte differentiation (Fig. 6). Therefore, dynamic interactions among OASIS, CREB4 and Luman regulate the spatio-temporal expression of Gcm1 during astrocyte differentiation. These OASIS family members activate UPR signaling in response to physiological ER stress to fine-tune the expression of Gcm1 followed by regulating astrocyte differentiation. UPR signaling activated by physiological ER stress is known to be crucial for the differentiation of secretory cells including osteoblasts (Murakami et al. 2009; Saito et al. 2011; Tohmonda et al. 2011), chondrocytes (Saito et al. 2009), and plasma cells (Gass et al. 2002; Iwakoshi et al. 2003). During the differentiation of progenitor cells into these mature secretory cells, secretory materials are gradually produced, and abundant nascent proteins are delivered to the ER. Such an event may serve as a trigger for physiological ER stress. Astrocytes also synthesize and secrete various neurotrophic factors and cytokines. Physiological ER stress caused by production of abundant secretory proteins could be activated during differentiation of NPCs into mature astrocytes. OASIS, CREB4 and Luman may be activated as transcription factors in response to this physiological ER stress. The difference between physiological and pathological ER stress remains unclear. Treatment of NPCs with low doses of several ER stressors, including tunicamycin and dithiothreitol, promotes the differentiation of NPCs into astrocytes but not apoptosis (Saito et al. 2012). Thus, physiological and pathological ER stress may be distinguished, in part, only by the difference of the burden levels on the ER. Additionally, recent studies have shown that ER stress transducers can mildly activate ER stress-independent phenomena such as changes in the lipid-constitution of the ER membrane (Volmer et al. 2013). It is possible that not only mild ER stress, which does not promote apoptosis, but also ER stress-independent phenomena may activate ER stress transducers in the same was as physiological ER stress during cell differentiation.

## Conclusion

**Fig. 7:**
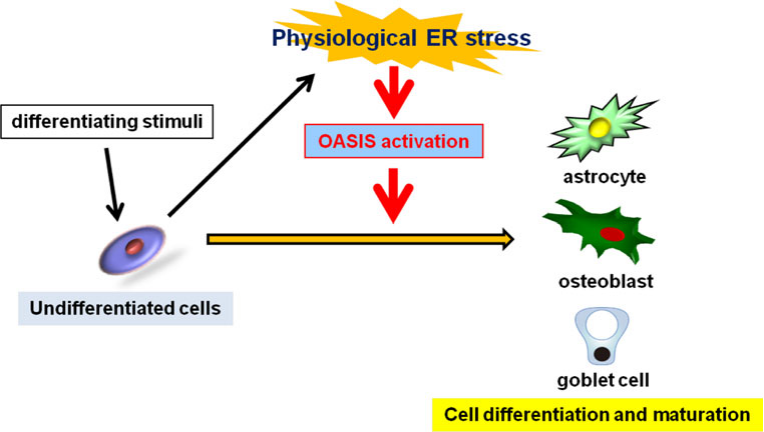
OASIS activated by physiological ER stress promotes various cell differentiation and maturation. Differentiation stimuli to undifferentiated cells initiates differentiation. Altered ER environments, such as the synthesis of abundant secretory proteins, causes physiological ER stress. Physiological ER stress activates OASIS followed by accelerating the differentiation and maturation of cells including astrocytes, osteoblasts and goblet cells

UPR signaling was originally found as a system for evading cellular damage in acute ER dysfunction. However, recent advanced studies have revealed that UPR signaling provides important signals for regulating cellular physiology. Not only canonical ER stress transducers, but also OASIS family members act as signaling centers for numerous networks originating from the ER. In particular, OASIS family members are specialized in biological regulation, including cell differentiation, maturation and maintenance of basal cellular homeostasis. Thus far, ER stress damages cells, and has been seen as a major cause of various diseases (Schroder and Kaufman 2005; Hotamisligil 2010). However, recent findings, as shown here, suggest that an appropriate ER stress provides an important signal for controlling cellular physiology. It would not be surprising that differentiation, maturation and functional regulations of other cells are similarly regulated by physiological ER stress. OASIS is involved in the differentiation of osteoblasts and goblet cells in the large intestine as well as in astrocytes (Murakami et al. 2009; Asada et al. 2012; Saito et al. 2012) (Fig. 7). Further, two previous reports regarding *Creb4* ^−/−^ mice revealed that this molecule plays a role in differentiation of sperm cells (Adham et al. 2005; Nagamori et al. 2006). BBF2H7 and Luman are involved in the differentiation of chondrocytes and DCs, respectively (Saito et al. 2009; Eleveld-Trancikova et al. 2010). CREBH, another OASIS family member, plays a role in activating expression of inflammatory response in the liver (Zhang et al. 2006). Besides OASIS family members, canonical ER stress transducers have also been defined to play roles in development, differentiation, and maturation of cells. IRE1-XBP-1 signaling is particularly important in cells that are active in protein secretion, such as antibody-secretory plasma cells, hepatocytes, and exocrine pancreatic cells (Calfon et al. 2002; Gass et al. 2002; Iwakoshi et al. 2003; Lee et al. 2005). PERK-eIF2α signaling mediates differentiation in insulin-secreting beta-cells and osteoblasts (Harding et al. 2001; Zhang et al. 2002; Wei et al. 2008; Saito et al. 2011). Thus, functional regulation mediated by physiological UPR signaling has been demonstrated to associate with various biological phenomena. Further studies including analyses on the detailed signaling and functions of the UPR components including OASIS family members are needed to better understand the diversity of the UPR system in mammals.

The signaling from the ER mediated by OASIS family members is presumed to be triggered by ER perturbation. However, is ER stress actually only the stimulation for activating OASIS family members that are required for initiating physiologically important signaling events? Indeed, OASIS is weakly expressed in hippocampal neurons under control conditions, although it is not activated by KA-induced ER stress (Chihara et al. 2009). These facts suggest that OASIS activation may be regulated by other cell-specific mechanisms as well as ER stress. Recently, it has been reported that ER stress transducers can be activated in response to ER stress-independent phenomena such as the changes in lipid-constitution of the ER membrane (Volmer et al. 2013). Further, OASIS does not have the ER stress-sensing domain in its ER luminal domain for activation. These facts indicate the possibility that OASIS family members, including OASIS, may be also activated by other mechanisms or signals including cell-specific differentiating stimuli, which is different from ER stress. Elucidation of the mechanisms responsible for their activation in specific tissues and cells may lead to clarification of the physiological functions of these OASIS family members. In addition, it is widely known that bZIP type transcription factors can form homodimers or heterodimers, and that this dimerization often affects their binding ability. As shown in this review, the OASIS N-terminus binds to CREB4 and Luman N-termini and forms heterodimers to fine-tune the expression of *Gcm1* in the CNS (Saito et al. 2012). ATF6 can bind to active forms of CREBH and serve as a potent enhancer to augment CREBH-mediated acute phase response gene transcription for the acute inflammatory response (Zhang et al. 2006). It is possible that changing the binding partners for each transcription factor may switch the target genes promoting expression, thus allowing fine-tuning of UPR signaling for regulating physiological phenomena. OASIS family members reveal unique cell- or tissue-specific expression patterns, but overlapping expression is also observed in each protein in the same cells or tissues. Thus, it is necessary to analyze the spatio-temporal hetero- or homo-dimerization of each protein in vivo to better understand the diversity of the UPR system in mammals.
